# Mesothelin-MUC16 binding is a high affinity, N-glycan dependent interaction that facilitates peritoneal metastasis of ovarian tumors

**DOI:** 10.1186/1476-4598-5-50

**Published:** 2006-10-26

**Authors:** Jennifer AA Gubbels, Jennifer Belisle, Masanori Onda, Claudine Rancourt, Martine Migneault, Mitchell Ho, Tapan K Bera, Joseph Connor, Bangalore K Sathyanarayana, Byungkook Lee, Ira Pastan, Manish S Patankar

**Affiliations:** 1 Department of Obstetrics and Gynecology, University of Wisconsin-Madison, Madison, USA; 2 Laboratory of Molecular Biology, National Cancer Institute, National Institutes of Health, Bethesda, MD, USA; 3 Department of Microbiology and Infectiology, Universite de Sherbrooke, Sherbrooke, Canada

## Abstract

**Background:**

The mucin MUC16 and the glycosylphosphatidylinositol anchored glycoprotein mesothelin likely facilitate the peritoneal metastasis of ovarian tumors. The biochemical basis and the kinetics of the binding between these two glycoproteins are not clearly understood. Here we have addressed this deficit and provide further evidence supporting the role of the MUC16-mesothelin interaction in facilitating cell-cell binding under conditions that mimic the peritoneal environment.

**Results:**

In this study we utilize recombinant-Fc tagged human mesothelin to measure the binding kinetics of this glycoprotein to MUC16 expressed on the ovarian tumor cell line OVCAR-3. OVCAR-3 derived sublines that did not express MUC16 showed no affinity for mesothelin. In a flow cytometry-based assay mesothelin binds with very high affinity to the MUC16 on the OVCAR-3 cells with an apparent K_d _of 5–10 nM. Maximum interaction occurs within 5 mins of incubation of the recombinant mesothelin with the OVCAR-3 cells and significant binding is observed even after 10 sec. A five-fold molar excess of soluble MUC16 was unable to completely inhibit the binding of mesothelin to the OVCAR-3 cells. Oxidation of the MUC16 glycans, removal of its N-linked oligosaccharides, and treatment of the mucin with wheat germ agglutinin and erythroagglutinating phytohemagglutinin abrogates its binding to mesothelin. These observations suggest that at least a subset of the MUC16-asscociated N-glycans is required for binding to mesothelin. We also demonstrate that MUC16 positive ovarian tumor cells exhibit increased adherence to A431 cells transfected with mesothelin (A431-Meso^+^). Only minimal adhesion is observed between MUC16 knockdown cells and A431-Meso^+ ^cells. The binding between the MUC16 expressing ovarian tumor cells and the A431-Meso^+ ^cells occurs even in the presence of ascites from patients with ovarian cancer.

**Conclusion:**

The strong binding kinetics of the mesothelin-MUC16 interaction and the cell adhesion between ovarian tumor cells and A431-Meso+ even in the presence of peritoneal fluid strongly support the importance of these two glycoproteins in the peritoneal metastasis of ovarian tumors. The demonstration that N-linked glycans are essential for mediating mesothlein-MUC16 binding may lead to novel therapeutic targets to control the spread of ovarian carcinoma.

## Background

The mucin MUC16 carries the peptide epitope CA125, which is a prominent molecular marker for monitoring the progression and recurrence of epithelial ovarian cancer (EOC) [1-3]. MUC16 is a very large mucin with an average molecular weight between 2.5–5 million Da [2,4]. The peptide backbone of MUC16 is composed of the N-terminal region, an extensive tandem repeat domain, and a C-terminal region with a short cytoplasmic tail [2]. The tandem repeat, a hallmark of all mucins, is composed of 18–60 repeats, each of which contains 156 amino acids [2]. The N-terminal domain has been proposed to be made up of 13,000 amino acids [4]. In addition to the extensive protein structure, MUC16 is also heavily glycosylated with both O-linked and N-linked oligosaccharides [5].

Enumeration of the basic structure of MUC16 has led to recent studies to define the biological properties of this mucin. The over expression of MUC16 by many tumors of epithelial origin suggest an important role for this mucin in tumorigenesis. We have demonstrated that MUC16 inhibits the cytolytic responses of human natural killer cells [6]. MUC16 also binds to galectin-1, a mammalian lectin that is expressed on human immune cells [7]. These studies suggest that one role of MUC16 is to prevent efficient anti-tumor immune responses.

Another postulated function of MUC16 is to facilitate cell-cell interactions. A recombinant fragment of MUC16 composed of the cytoplasmic region and a partial tandem repeat domain binds to mesothelin, a glycoprotein that is highly expressed by ovarian tumors and mesotheliomas [8,9]. Mesothelin is normally expressed on the mesothelial cells lining the peritoneal cavity [8]. In a heterotypic cell adhesion assay, the epithelial ovarian tumor cells, OVCAR-3, bind to the mesothelin expressing murine endothelial-like cell line, LO [9]. Addition of increasing concentrations of an anti-mesothelin antibody inhibited the binding between these cells. These experiments suggest that mesothelin may play an important role in the metastasis of the ovarian tumors within the peritoneum.

Mesothelin is a 40 kDa glycoprotein that is initially synthesized as a 70 kDa precursor [10]. Proteolytic cleavage results in the release of a 32 kDa fragment called the megakaryocyte potentiating factor [11]. The 40 kDa fragment of this precursor is anchored to the cell surface by a glycosylphosphatidyl inositol linkage and is referred to as mesothelin [8]. Initial experiments suggested a role for mesothelin in cell adhesion events [8]. However, since mesothelin knock-out mice do not exhibit any adverse pathology, the exact function of this molecule is not clear [12].

Considering the potential importance of the mesothelin-MUC16 interaction in ovarian tumor metastasis within the peritoneum, we have performed additional studies to carefully define its molecular characteristics. The data presented conclusively shows that mesothelin binds to native MUC16 expressed by the ovarian cancer cell line OVCAR-3 and also to MUC16 isolated from the peritoneal fluid of patients with EOC.

Here we provide firm evidence that N-linked oligosaccharides of MUC16 are required for the binding of this mucin to mesothelin. Kinetic analysis is provided to demonstrate that mesothelin-MUC16 binding is a high affinity interaction that increases cell adhesion between MUC16 and mesothelin expressing cells. The importance of this observation to the peritoneal metastasis of ovarian tumors is discussed.

## Results

### Mesothelin binds to native MUC16

**Figure 1:**
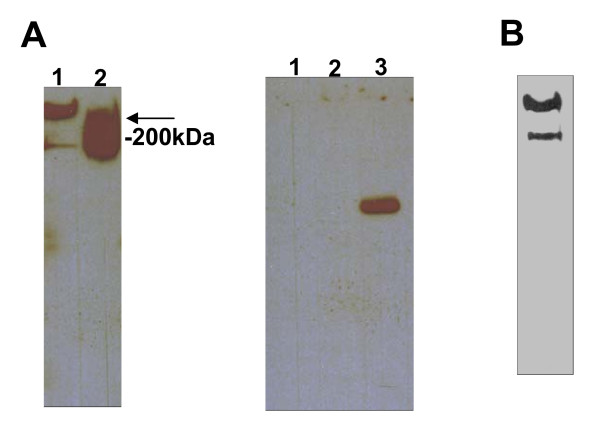
**Mesothelin binds to native MUC16**. (A) Meso-Fc binding to MUC16 samples from the ascites of patient #2 (100 U of CA125; lane 1) and from OVCAR-3 cells (500 U of CA125; lane 2) is shown (left panel). A control blot (right panel) with MUC16 from patient #2 (100 U of CA125; lane 1) and OVCAR-3 (500 U of CA125; lane 2) and meso-Fc (Lane 3) was overlaid with secondary antibody only. (B) For comparison, MUC16 from OVCAR-3 (100 U of CA125) was detected by VK-8 antibody. Note that since only 100 U (CA125) of OVCAR-3-derived MUC16 was loaded the banding pattern is identical to that of lane 1 of (A).

Although mesothelin has been previously shown in direct assays to bind a recombinant fragment of MUC16 [9], it was important to conclusively demonstrate this interaction with native forms of this mucin. MUC16 was partially purified, by size exclusion chromatography, from OVCAR-3 cell culture media and from the peritoneal fluid of EOC patients as described in our earlier report [5]. These MUC16 samples showed strong binding to the recombinant mesothelin tagged with the Fc fragment of rabbit IgG (meso-Fc) in western blot overlay experiments (Figure 1a). The banding pattern observed after overlaying the membranes with meso-Fc was identical to that obtained in blots where the mucin was detected with the anti-MUC16 antibody, VK-8 (Figure 1b) [13,14]. We also determined that this observation was not an artifact of direct binding of the Fc portion to MUC16, as binding of purified rabbit Fc to the mucin could not be detected by western blotting (data not shown).

**Figure 2:**
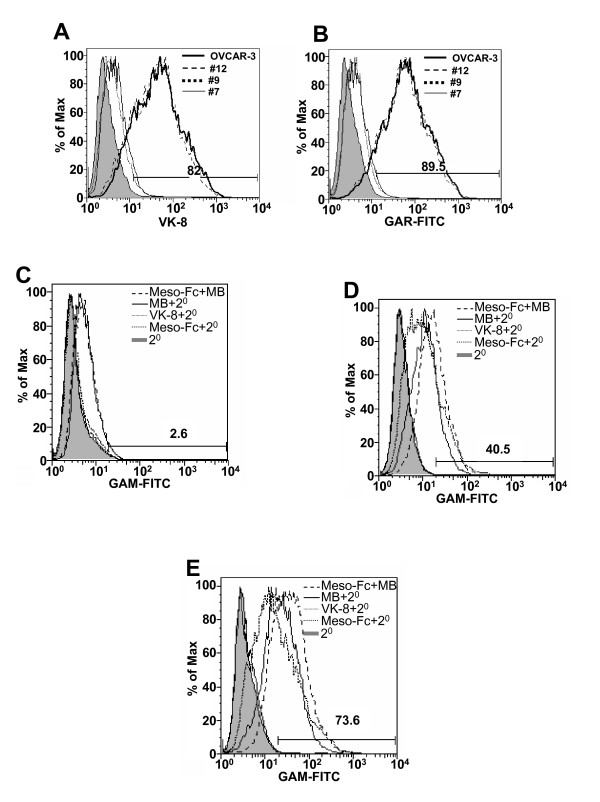
**Mesothelin primarily binds to MUC16 on the OVCAR-3cell surface**. (A)MUC16 expression on the parental OVCAR-3 and the sublines #7, #9, and #12 was determined by flow cytometry using the VK-8 antibody. (B) Meso-Fc binding to the OVCAR-3 cells and the sublines #7, #9, and #12 was determined by flow cytometry using a GAR-FITC reporter antibody. (C-E) The MB antibody was used to determine meso-Fc binding to #7 (C), #9 (D), and #12 (E) and compared to expression of native mesothelin and MUC16 on the surface of these cells.

In our initial flow cytometry studies we observed that meso-Fc would bind strongly to the OVCAR-3 cell surface. Arguably, meso-Fc could bind to other ligands on the OVCAR-3 surface in addition to MUC16. To determine the specificity of meso-Fc binding to MUC16 on the cell surface, we utilized stable sublines derived from the parental OVCAR-3 cell line that did not carry MUC16 on their cell surface. These MUC16 "Knock-down" cells, designated #7 and #9, express an intracellular scFv fragment of the VK-8 antibody that captures the nascent MUC16 molecules in the endoplasmic reticulum, thereby inhibiting the expression of this mucin on the cell surface (Pinard et al, manuscript submitted). The #7 and #9 MUC16 knock-down cells showed only minimal affinity for meso-Fc (Figure 2a). Control cells designated as #12, which express a scFv fragment of a non-specific murine IgG, exhibited strong binding to meso-Fc (Figure 2b). These results indicated that MUC16 was the only ligand for meso-Fc on the OVCAR-3 cell surface. Similar results were obtained (figure 2c–e) when the binding of meso-Fc to these sublines was detected by using the anti-mesothelin antibody MB [15].

The #9 cells upon continuous culture (usually after 6 passages) are "leaky" and may sometimes express higher amounts of MUC16 (Figure 2d). Such an increased expression of MUC16 on the #9 cells correlated with enhanced meso-Fc binding to these cells (Figure 2d).

### Mesothelin binds to MUC16 with strong affinity

**Figure 3:**
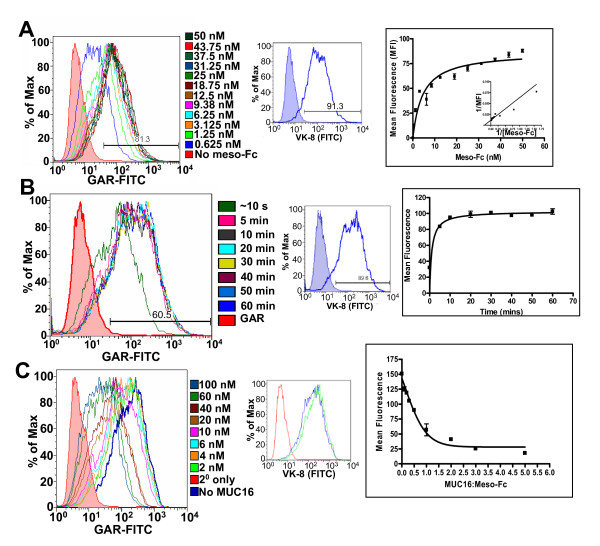
**Kinetics of the mesothelin-MUC16 interaction**. (A) Binding of specified amounts of meso-Fc to OVCAR-3 was detected by flow cytometry. The left panel shows data for one representative experiment. Composite analysis of three independent experiments is shown in the panel on the right. Middle panel shows native expression of MUC16 on the OVCAR-3 cells used in this assay. (B) Time kinetics of meso-Fc (25 nM) binding to OVCAR-3 cells was determined by flow cytometry. After incubation with meso-Fc for the designated time intervals, the cell suspensions were diluted with 2 ml of buffer (a step that takes approximately 10 s) and analyzed. Raw flow cytometry data from one experiment is shown in panel on the left and composite analysis of two independent experiments is in the right panel. The middle panel shows expression of MUC16 on the OVCAR-3 cells used in this experiment. (C) Inhibition of meso-Fc binding to OVCAR-3 cells by soluble MUC16 was also measured by using a flow cytometry assay. Meso-Fc preincubated with designated amounts of MUC16 was added to the cells. Binding was analyzed by flow cytometry. Left panel shows raw data for one experiment and panel on right shows composite data of two independent assays. The middle panels show VK-8 binding to the OVCAR-3 cells used in this experiment in the presence (green) or absence (blue) of soluble MUC16 (100 nM).

Since MUC16 was shown to bind to mesothelin on the surface of the OVCAR-3 cells, we utilized this specificity of binding to devise a flow cytometry based assay to define the affinity and kinetics of the mesothelin-MUC16 interaction. In the first experiment, the OVCAR-3 cells were incubated with increasing amounts of MUC16 for 1 h prior to analysis by flow cytometry. Meso-Fc binding to the OVCAR-3 cells was concentration dependent with an apparent dissociation constant (K_d_) of 5 nM (Figure 3a). Since the cell surface expression of MUC16 increases with the amount of time that the OVCAR-3 cells are maintained in culture, their capacity to bind meso-Fc amplifies proportionately (data not shown). This effect results in slight variations in the binding kinetics of meso-Fc. On average, the K_d _values range between 5–10 nM.

We routinely incubated the OVCAR-3 cells with meso-Fc for 1 h to detect binding of this chimera to the cell surface. In a time course study we have shown, however, that the optimum binding of meso-Fc to the OVCAR-3 cell surface is achieved within 5 mins (Figure 3b). Significant binding of meso-Fc was observed even after an approximately 10 s incubation of this chimera with the OVCAR-3 cells. Together these experiments suggest that the mesothelin-MUC16 binding is a very high affinity interaction that occurs with rapid kinetics.

Inhibition studies using soluble MUC16 at half the molar amount of meso-Fc reduced the binding of this chimera to the OVCAR-3 cells by approximately 80% (Figure 3c). However, residual binding of meso-Fc to the cell surface is always observed even when a 5-fold molar excess of MUC16 is used in these experiments (Figure 3c).

### N-linked oligosaccharides of MUC16 are required for mesothelin binding

**Figure 4:**
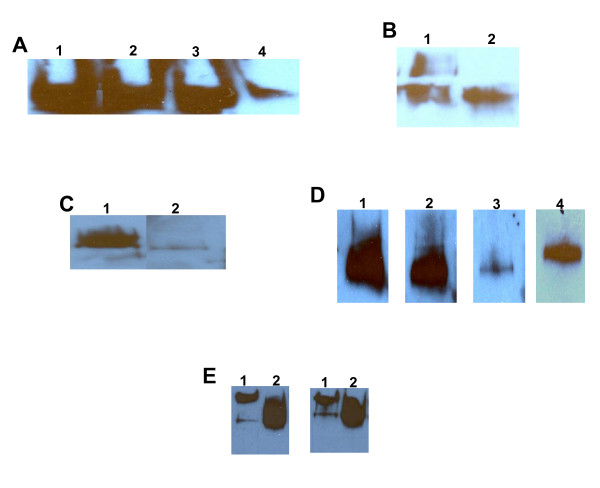
**Mesothelin recognizes oligosaccharides expressed on MUC16**. (A) Meso-Fc binding to OVCAR-3-derived MUC16 (200 U CA125/lane) after oxidation with 1 mM (lane 2) or 10 mM (lane 4) SMP was detected in overlay experiments. Matching controls are in lanes 1 and 3, respectively. The blots were sequentially overlaid with meso-Fc, MN and a horseradish peroxidase conjugated secondary. (B) Meso-Fc binding to desailylated (lane 2) and non-desialylated (lane 1) MUC16 (150 U CA125/lane) from OVCAR-3 cells was determined in overlay experiments. (C) Meso-Fc binding to MUC16 (200 U CA125/lane) treated with PNGaseF (lane 2) or with buffer only (lane 1) was determined by Western blot overlay experiments. (D) Inhibition of bacterial mesothelin binding to OVCAR-3-derived MUC16 (200 U of CA125/blot) by ConA (blot 2), WGA (blot 3), and E-PHA (blot 4) detected in overlay experiments (D). No lectin was added in blot 1. (E) MUC16 from patient #2 (lane 1; 100 U of CA125) or from OVCAR-3 (lane 2; 500 U of CA125) were overlaid with meso-Fc in the presence (blot on left) or the absence (blot on right) of 0.5 M α-methylmannopyranoside. MN was used to detect meso-Fc binding in all experiments. Since full gel profiles have been shown in Fig. 1, only partial blots are shown here.

Considering its mucinous nature we next investigated if mesothelin recognized the protein of the oligosaccharide chains of MUC16. Periodate treatment of MUC16 under conditions that selectively oxidize only the vicinal hydroxyl groups present on the C8–C9 positions of terminal sialic acids [16,17] had no effect on the binding of meso-Fc to the mucin (Figure 4a). This result was supported by the observation that MUC16, upon desialylation with neuraminidase, retained its ability to bind to meso-Fc (Figure 4b). On the contrary, oxidation of the vicinal hydroxyl groups present on the terminal sialic acids as well as the internal monosaccharides of the entire glycan chains of MUC16 resulted in an almost complete loss of meso-Fc binding to MUC16 (Figure 4a). MUC16 is extensively glycosylated with both O-linked and N-linked glycans [5]. The N-linked oligosaccharides were selectively removed by treating MUC16 with peptide-N-glycosidase (PNGaseF). Enzymatic removal of the N-linked glycans corresponded with a decrease in molecular weight of MUC16 as detected by western blot analysis (data not shown). PNGaseF treated MUC16 almost completely lost its ability to bind to meso-Fc (Figure 4c).

We next tested a panel of lectins for their ability to interfere with the binding of mesothelin to MUC16. Since the lectins could potentially bind to oligosaccharide chains expressed on meso-Fc and hence complicate the analyses, we employed recombinant non-glycosylated mesothelin that was synthesized by bacterial cells. In western blot overlay experiments, the lectin wheat germ agglutinin (WGA) strongly inhibited the binding of bacterial mesothelin to MUC16 (Figure 4d). Appreciable inhibition was also observed with the lectin erythroagglutinating phytohemagglutinin (E-PHA), however, concanavalin A (ConA) was unable to block this binding (Figure 4d).

**Figure 5:**
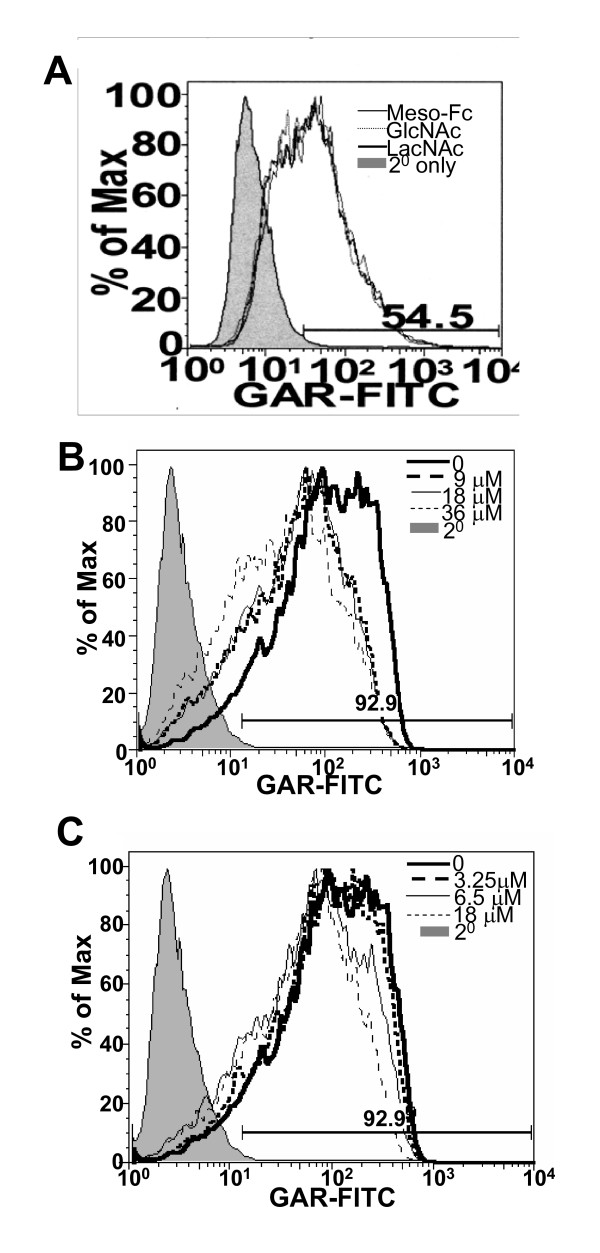
**Effect of glycoconjugates on mesothelin-MUC16 interaction**. (A) Binding of meso-Fc to OVCAR-3 cells was measured in the presence of 0.25 mM N-acetylglucosamine (GlcNAc) or N-acetyllactosamine (LacNAc) by flow cytometry. (B and C) Similarly the effect of ovomucoid (B) and ovotransferrin (C) on meso-Fc binding to OVCAR-3 was measured using the same technique. Meso-Fc binding was monitored by using the GAR-FITC reporter antibody.

The ConA ligand α-methyl mannopyranoside, does not inhibit the binding of meso-Fc to MUC16 in western blot overlay experiments (Figure 4e) and neither do the WGA ligands N-acetylglucosamine and N-acetyllactosamine [18,19] (Figure 5a). Chicken egg glycoproteins, ovotransferrin (conalbumin) and ovomucoid, that express the E-PHA ligands bi- and tri-antennary bisecting type N-glycans [20,21] weakly inhibited meso-Fc binding to the OVCAR-3 cells (Figure 5b,c). The oligosaccharide sequences recognized by WGA and E-PHA are also expressed on human erythrocytes although this cell type does not carry MUC16. Meso-Fc however, did not bind to the erythrocytes (Additional File 1). We also determined that the #7 and the #9 cells bound to WGA and E-PHA to the same extent as the #12 and the parental OVCAR-3 cells (Additional File 1). The #7 and #9 sublines cells have no affinity for meso-Fc, however (Figure 1b).

A protein sequence analysis suggested that mesothelin is primarily composed of helix-turn-helix repeats. The β-pleated sheets that typically compose the C-type and I-type lectins are not present in mesothelin. We have also confirmed the earlier observation [9] that meso-Fc binding to MUC16 is independent of divalent cations (data not shown).

### Cell adhesion mediated via MUC16-mesothelin interaction

**Figure 6:**
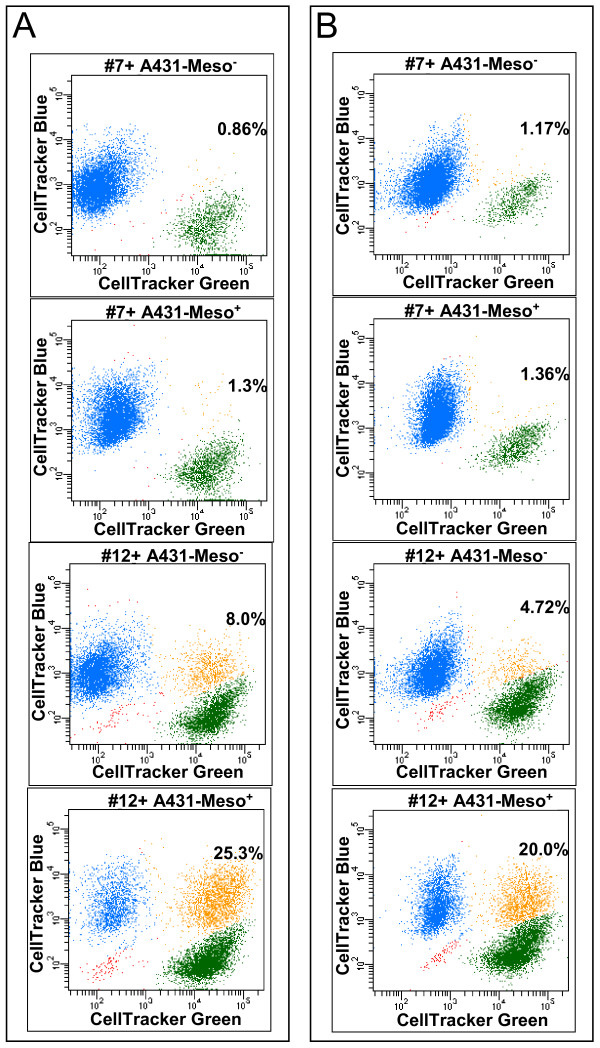
**MUC16 expressing ovarian tumor cells form conjugates with mesothelin positive cells**. (A) CellTracker green labeled sublines #12 and #7 were coincubated with either A431-Meso^+ ^or A431-Meso^- ^cells in PBS containing 1% BSA. The heterotypic doublets formation was measured by flow cytometry. The #7 and #12 sublines are represented in green, the A431 cells are in blue and the heterotypic doublets are depicted in orange. The percentages of all live cells that form heterotypic doublets are shown for each plot. Cell debris is in red. (B) Heterotypic doublet formation between the sublines #12 and #7 and the A431 cells in the presence of ascites from patient#15 is shown. The sublines are represented in green and the A431 cells are in blue. Heterotypic doublets are in orange. The percentage of heterotypic doublets is shown for each plot. Cell debris is in red.

Interaction between human MUC16 and murine mesothelin was shown to mediate cell adhesion between the OVCAR-3 and the LO cells, a murine cell line [9]. We therefore decided to demonstrate if interaction between human MUC16 and human mesothelin would also mediate cell binding. For this purpose we utilized the #12 and the #7 sublines along with human epidermoid epithelial A431 cells expressing recombinant mesothelin (A431-Meso^+^). To obtain quantitative data we performed a flow cytometry assay where the formation of heterotypic doublets was determined. The ovarian tumor cells and the A431 cells were labeled with CellTracker Green and CellTracker Blue dyes, respectively. Heterotypic doublet formation between the green and blue labeled cells was measured by flow cytometry. When the #12 and the A431-Meso^+ ^cells were co-incubated, 25% of the total live cells formed heterotypic doublets (Figure 6A; Additional File 2). The extent of heterotypic doublet formation was only 1.3% between the #7 and the A431-Meso^+ ^cells. Very small numbers (0.8%) of doublets were observed between the #7 and the A431-Meso^- ^cells. The #12 and the A431-Meso^- ^combination produced approximately 8% doublets suggesting that MUC16 may mediate cell adhesion via mesothelin-independent mechanisms.

The ovarian cancer cells sloughing off from the tumor are bathed in peritoneal fluid that contains high amounts of MUC16 ([22]; Patankar, Belisle, and Gubbels, unpublished observation). We therefore determined if the heterotypic doublet formation could occur even in the presence of peritoneal fluid from EOC patients (#15 and #24). Although the presence of ascites reduced the heterotypic cell adhesion between the #12 and the A431-Meso^+ ^cells by approximately 33%, robust doublet formation was still observed (Figure 6B; Additional File 2). The ascites fluid from patients #15 and #24 had CA125 levels of 45,600 U/ml and 83,600 U/ml. These values for CA125 correspond to 7 and 13 nM concentration of MUC16, respectively (see Discussion). Since we routinely observe that the concentration of CA125 in the peritoneal fluid is between 20,000–100,000 U/ml the ascites samples we have used in this study are highly representative.

**Figure 7:**
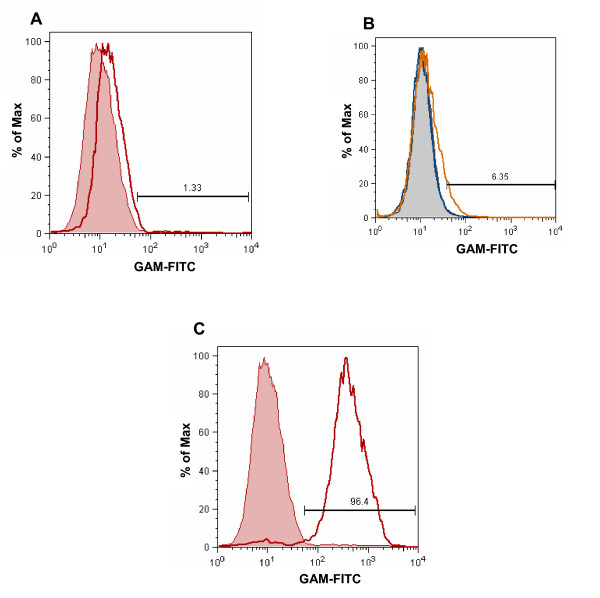
**Soluble MUC16 has a lower affinity for mesothelin**. (A) A431-Meso^+ ^cells were incubated with purified MUC16 (50,000 U/ml). After 1 h, the amount of MUC16 on the cell surface was measured using the VK-8 antibody and a FITC labeled secondary. (B) Binding of A431-Meso^+ ^cells to MUC16 from peritoneal fluid was measured by flow cytometry. The cells were cultured in the peritoneal fluid from patient #15 that contains (84,000 U of CA125/ml). MUC16 binding was detected using the VK-8 and the FITC-labeled secondary antibodies. (C) The A431-Meso^+ ^cells were labeled with MN and a FITC-conjugated secondary. Mesothelin expression was measured by flow cytometry.

Considering the relatively small effect of soluble MUC16 from the ascites on heterotypic doublet formation, we further investigated the ability of the A431-Meso^+ ^cells to bind soluble MUC16. In this experiment, the A431-Meso^+ ^cells were incubated with either purified MUC16 or with ascites from EOC patient #15. Following incubation for 1 h, the amount of MUC16 on the A431-Meso^+ ^cells was measured by flow cytometry. Only a slight increase in cell surface MUC16 was detected which was not proportional to the amount of mesothelin expressed on these cells (Figure 7).

**Figure 8:**
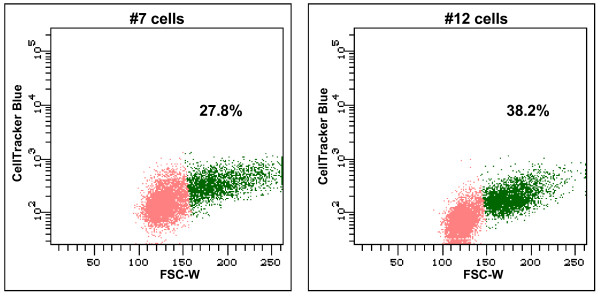
**MUC16-Mesothelin binding increases spheroid formation in ovarian tumor cells**. The #7 and the #12 sublines were labeled with CellTracker Green. After keeping the cells in suspension for 1 h in peritoneal fluid, the homotypic doublet formation was measured by flow cytometry. The singlet events are shown in pink and the doublets are in green. The percentage of live cells that form the homotypic doublets is shown for each plot.

Finally, we also determined that the #12 cells were able to form a higher number of homotypic doublets as compared to the #7 cells (Figure 8). The mean of seven independent experiments indicated that the homotypic doublets for the #12 and the #7 cells were 35.2% and 26.7% (P < 0.0001) of the total live cells analyzed. The interaction of mesothelin and MUC16 may enhance the physical association of tumor cells and contribute to spheroid formation. These homotypic doublets were observed even in the presence of peritoneal fluid (Figure 8). The A431-Meso^+ ^cells upon incubation with MUC16 did not exhibit any appreciable homotypic doublet formation and neither did they bind to meso-Fc (data not shown). This data is consistent with the observation that the A431-Meso^+ ^cells do not bind appreciable amounts of MUC16 (Figure 7).

## Discussion

In a previous report, mesothelin was clearly shown to be a binding partner for MUC16 [9]. Cell binding studies suggested that mesothelin-MUC16 interaction could facilitate peritoneal metastasis of ovarian tumors. This hypothesis is also supported by the observation that both mesothelin and MUC16 are co-localized in immunohistochemical studies of ovarian tumors. Understanding the importance of MUC16-mesothelin binding may lead to novel therapies to control the peritoneal spread of ovarian tumors. With this in mind, a high throughput assay has been developed to determine the ability of biological agents to inhibit ovarian tumor metastasis [23].

To this date, the biochemical basis of the MUC16-mesothelin binding is not clearly understood. Our data addresses this deficiency. Here, we have provided evidence that mesothelin has a very strong affinity for MUC16 with an apparent K_d _of approximately 5 nM. The binding of these two molecules occurs with rapid kinetics as demonstrated by our flow cytometry based assays. We have conclusively shown that mesothelin interacts with both soluble and cell surface associated forms of native MUC16.

For the first time, we demonstrate that the N-linked glycans of MUC16 are required for binding to mesothelin. In this context it is important to note that Rump et al have reported that inhibition of O-glycosylation of the OVCAR-3 cells reduced the binding of mesothelin to the cells [9]. Since no data was provided to support this observation it is not possible to directly compare the results of this study with our observations. The importance of MUC16 N-glycans in the binding to mesothelin suggests at least two possibilities. First, the removal of the oligosaccharides may result in perturbation of the mesothelin binding site on the MUC16 protein backbone. This suggestion is albeit difficult to conceptualize since the MUC16 used in our western blot overlay experiments was already denatured. The second possibility that is currently under investigation in our laboratory is that mesothelin acts as a lectin and directly binds to at least a subset of the MUC16 associated N-glycans. If true, the inhibition of the mesothelin-MUC16 binding by WGA and E-PHA suggests that polylactosamine or bisecting type N-linked glycans may be the potential ligands for mesothelin [18,19,24-26]. The weak inhibition of mesothelin binding to OVCAR-3 cells by ovomucoid and ovotransferrin supports the involvement of the bisecting type N-glycans. Another possibility is that appropriate presentation of oligosaccharide ligands is required for mesothelin binding. WGA and E-PHA binding sites are abundant on human erythrocytes (Gubbels and Patankar, unpublished results) and the #7 and #9 cells (Additional File 1). Yet, meso-Fc has no affinity for these cells. We propose that precise positioning and/or expression of appropriate glycans in high density is required for mesothelin to bind to candidate glycoprotein partners. MUC16, as a mucin, is extensively glycosylated. These glycans are likely to be presented in a repeating array on the tandem repeat domain of MUC16 making it a very high affinity mesothelin ligand.

Exhaustive studies that involve glycomic analysis of mesothelin bound MUC16 fragments will be required to clearly define these oligosaccharide ligands. It should be noted however, that mesothelin does not carry any of the classical carbohydrate recognition domains that are found in a majority of the mammalian lectins. Thus if mesothelin directly binds to oligosaccharide ligands it may do so via an as yet unidentified carbohydrate binding peptide epitope.

High concentrations of soluble MUC16 are present in the peritoneal fluid of EOC patients ([22]; Patankar, Belisle, and Gubbels, unpublished observations). Since the soluble MUC16 may inhibit mesothelin-MUC16 binding, the proposal that this interaction facilitates peritoneal metastasis of ovarian tumors seems counter-intuitive. However, having analyzed more than 30 samples of ascites derived from individual EOC patients we have observed that the concentration of CA125 in this fluid ranges from 20,000–100,000 U/ml. Since the most highly purified MUC16 samples have a CA125 specific activity of 2.5 × 10^6 ^U/mg of total protein and assuming that the average molecular weight of MUC16 is 3 × 10^6 ^Da, these values of CA125 in the ascites correspond to MUC16 concentrations of 1–10 nM. Inhibition studies indicate that MUC16 even when used at 100 nM concentration was unable to completely block the binding of meso-Fc to the OVCAR-3 cells (Figure 3c). Furthermore, there is a distinct possibility that soluble MUC16 may have a lower affinity for mesothelin than the cell surface associated counterpart of this mucin. This is highlighted by our demonstration that A431-Meso^+ ^cells bind to only minor amounts of soluble MUC16 (Figure 7). The proteolytic processing that occurs when the MUC16 is shed from the surface of the tumor cells or subsequent digestion of the N-linked glycans by glycosidases in the peritoneal fluid may alter the soluble MUC16 and make it a less effective ligand for mesothelin. The lower affinity of mesothelin for soluble MUC16 also makes it less likely that the mucin can act as a cross-linking agent by attaching to mesothelin expressed on the mesothelial and the ovarian tumor cells.

Binding of the tumor cells to the mesothelium via the mesothelin-MUC16 interaction may provide a necessary first step for metastasis. In this respect, the binding of mesothelin to the MUC16 glycans may be akin to the initial binding of leukocytes to the inflamed endothelium that is mediated by the selectins [27]. Tumor cell attachment to the mesothelium may by itself not provide the strong adhesive force required to maintain this heterotypic adhesion. Instead, initial binding via this mechanism may lead to recruitment of strong adhesive events mediated by CD44, β-1 integrins, and other cell adhesion molecules [28-30]. *In vitro *binding of ovarian cancer cells to the mesothelium is only partially inhibited by anti-CD44 and anti-β-1 integrin antibodies [28,29]. It remains to be demonstrated if simultaneous utilization of antibodies directed against mesothelin, CD44 and β-1 integrins will substantially inhibit binding of ovarian tumor cells to the mesothelium.

Mesothelin, like MUC16, is overexpressed by the ovarian tumor cells [31]. Co-expression of both these molecules may lead to further recruitment of additional tumor cells to the site of metastasis. Thus, the tumor load at the site of secondary metastasis may not only increase due to the uncontrolled expansion of the cancer cells, but also by the binding of additional tumor cells that are sloughed off from the primary or secondary tumor sites. In this context it is also important to note that ovarian cancer cells form multicellular spheroids in the peritoneal fluid [32,33]. Our experiments demonstrating significantly higher homotypic adhesions between the #12 cells as compared to the MUC16 negative #7 cells (Figure 8) supports this proposal.

**Figure 9:**
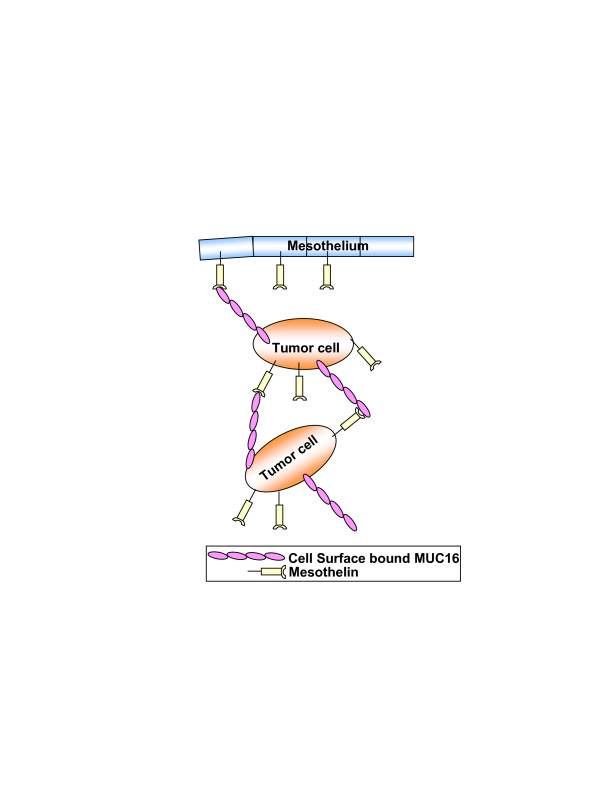
**Model for peritoneal metastasis of ovarian tumors**. A model showing the importance of MUC16-mesothelin interaction in the peritoneal metastasis of ovarian tumors is shown.

A model showing the role of mesothelin-MUC16 interaction in the peritoneal metastasis and ovarian tumor aggregate is shown in Figure 9. Granted, other mechanisms such as CD44 interactions are also important while studying the metastasis of ovarian tumors. However, since their relationship with the mesothelin-MUC16 interactions is not understood, these mechanisms are not depicted in the proposed model.

## Conclusion

In conclusion, we have provided new data on the interaction between two glycoproteins, mesothelin and MUC16, whose expression is highly upregulated by epithelial ovarian tumors. The high affinity and specificity of this binding makes these two molecules excellent therapeutic targets in the effort to prevent and control the peritoneal metastasis of epithelial ovarian cancer. We will make continued efforts to determine the carbohydrate ligand on MUC16 that is required for mesothelin binding and will further investigate the biological function and significance of these two interacting glycoproteins.

## Methods

### Cells, antibodies and other reagents

The anti-MUC16 antibodies OC125 and VK-8 were obtained from Dr. Robert Bast and Beatrice Yin, respectively. Anti-mesothelin antibodies MN and MB were obtained as described previously [15]. Lectins were from E-Y Laboratories, and all other regents were purchased from Sigma of Fisher unless otherwise noted.

The OVCAR-3 cells were purchased from ATCC (Rockville, MD). The OVCAR-3 derived CA125 knockdown stable sublines (#7 and #9) or control subline (#12) expressing an anti-CA125 scFv fragment or nonspecific scFv, respectively, were generated as described elsewhere (Pinard et al, manuscript submitted). Blasticidin (0.5–lμg/ml) was added in the media of the stable sublines to maintain scFv expression. The A431-Meso^+ ^and A431-Meso^- ^cells were obtained as described previously [34].

### Isolation of native MUC16

MUC16 from OVCAR-3 cells was isolated as described in our previous study [5]. MUC16 from ascites samples was isolated as follows. Ascites from ovarian cancer patients who had consented to participate in this study, was obtained using standard surgical protocols. The protocols for obtaining and processing of the ascites fluid samples were approved by the Institutional Review Board. The ascites fluid was centrifuged to remove cellular debris and then stored at -20°C until use. Ascites (500 ml) was concentrated 5-fold in an Amicon concentrator fitted with a 100 kDa ultrafiltration membrane. The concentrated ascites was separated in batches on a Sephacryl-500 HR column. The column was eluted with 10 mM ammonium bicarbonate containing 0.02% sodium azide. Fractions were monitored for absorbance at 280 nm and the void peak that contained the majority of the MUC16 was pooled and purified once again on a second Sephacryl 500-HR column. The void peak was again pooled and concentrated. The total protein in this peak was measured by using the BCA (Pierce) reagent. The number of units of CA125 in the sample was determined by using the standard clinical radioimmunoassay. The specific activity of MUC16 derived from OVCAR-3 cells and the ascites was approximately 1–2 × 10^6 ^and 5 × 10^5 ^U of CA125/mg of total protein, respectively.

### Preparation of recombinant mesothelin proteins

Recombinant bacterial mesothelin and mesothelin-Fc fusion proteins were prepared as previously described [34]. Briefly, the extracellular domain of human mesothelin (GenBank accession number AY743922) was expressed as a fusion protein with maltose-binding protein (MBP) using a modified pMAL-p2X vector (NEB, Beverly, MA) containing the tobacco etch virus (TEV) cleavage site. The fusion protein was directed to periplasm of *Escherichia coli*. After TEV cleavage (Invitrogen, Carlsbad, CA), the samples were applied to an amylase column for removal of MBP. The same extracullular domain of mesothelin was also expressed as a fusion protein with Fc using a modified pSecTag2 vector (Invitrogen, Carlsbad, CA) containing rabbit IgG Fc. The plasmid was transfected into HEK 293T cells with LipofectAMINE (Invitrogen). The mesothelin-Fc proteins were harvested from the culture supernatant and purified with a Hi-trap protein A column (GE Healthcare). The purity of both bacterial mesothelin and mesothelin-Fc fusion proteins was over 95%.

### Western blot overlay experiments

MUC16 was separated on 10% SDS-PAGE gels and electroblotted on a PVDF membrane. The membranes were blocked for 1 h at 25°C with Tris buffered saline (TBS; 20 mM Tris containing 150 mM sodium chloride) containing 0.5% Tween 20 and 3% bovine serum albumin (BSA). The membrane was then overlaid overnight at 25°C with 0 or 1.9 μg of meso-Fc or recombinant bacterial mesothelin in 10 ml of TBS containing 0.5% Tween 20 and 1.5% BSA. The membranes were washed thoroughly with TBS containing 0.5% Tween 20 and sequentially overlaid with the anti-mesothelin antibodies (1:20 dilution) and horseradish peroxidase labeled goat anti-mouse IgG (Pierce; 1:20,000 dilution).

In some experiments following blocking with BSA, the membranes were incubated with biotinylated lectins (10 μg/ml; purchased from E.Y. Laboratories) for 2 h at 25°C. Following this incubation, the membranes were probed sequentially with recombinant bacterial mesothelin, and binding was detected using the MN antibody. To demonstrate that the lectins used in this study bind to MUC16, the blots were blocked with BSA and overlaid with the respective biotinylated lectins. Binding of the lectins to MUC16 was detected using peroxidase labeled avidin.

### Chemical and enzymatic modification of the MUC16 oligosaccharide chains

Oxidation of the MUC16 oligosaccharides by sodium metaperiodate (SMP) was carried out as described previously [16,17]. Oxidation of the terminal sialic acids was achieved by treatment of MUC16 with 1 mM SMP for 10 min at 4°C. To oxidize the vicinal hydroxyl groups in the entire glycan chain, MUC16 was treated with 10 mM SMP for 1 h at 25°C. Following oxidation the excess SMP was neutralized by adding 10-fold molar excess of glycerol. In matching controls the SMP was neutralized with glycerol prior to addition to treatment with the mucin. Finally, the test and control MUC16 samples were treated with 100 mM sodium borohydride.

MUC16 (500 U of CA125) was resuspended in 50 mM sodium acetate buffer pH 5.5 in a total volume of 50 ml. To this solution was added 1 U of type X neuraminidase from *Clostridium perfringens *and the digestion was conducted overnight at 37°C. A matching control sample of MUC16 was incubated in the reaction buffer under identical conditions, except that no neuraminidase was added. Digestion with neuraminidase was performed in duplicate. To determine meso-Fc and VK-8 binding, MUC16 corresponding to 150 U of CA125 was analyzed by Western blotting.

Removal of N-linked glycans was achieved by digesting MUC16 (1000 U of CA125) with PNGaseF (Glyko). Reaction buffers supplied by the manufacturer along with the enzyme were used for this digestion. The reaction mixture was incubated overnight at 37°C. Matching control was treated under identical conditions, except that PNGaseF was not added. Test and control MUC16 (250 U of CA125) was used for Western blot analysis.

### Meso-Fc binding to cells by flow cytometry

To determine binding to the cell surface, the cells (1 × 10^6^) were incubated with the designated concentration of meso-Fc, on ice for 1 h. In most experiments, meso-Fc bound to the cells was detected using a FITC-conjugated GAR secondary antibody (1:1000 dilution). In some experiments, after incubation with meso-Fc, the cells were washed and incubated with MB (1:20 dilution) followed by a FITC-labeled GAM secondary antibody. The cells were analyzed on a FACSCalibur benchtop flow cytometer (Becton Dickinson).

For the time kinetics, the OVCAR-3 cells were incubated with meso-Fc (25 nM) for the designated time intervals. Inhibition studies were conducted by incubating meso-Fc (25 nM) with designated concentrations of either, MUC16, N-acetylglucosamine, N-acetyllactosamine, ovomucoid, or ovotransferrin for 1 h at 4°C. OVCAR-3 cells (2.5 × 10^5^) were added and allowed to incubate in this mixture for 1 h at 4°C. FITC-conjugated GAR secondary antibody was used to detect meso-Fc binding to the cell surface in all of these experiments. Following flow cytometry, the mean fluorescence was determined and plotted using the GraphPad Prism software package. The K_d _and B_max _values from the non-linear regression were used for the Lineweaver-Burke plot.

#### Lectin binding experiments

The #12, #9, and #7 cells (500,000) were incubated with 4.5 μg/ml of biotinylated WGA and E-PHA PBS-BSA (100 μl). After incubation at 4°C for 1 h the cells were washed twice with PBS-BSA and labeled with FITC-conjugated avidin. After 30 min incubation, the cells were washed and analyzed by flow cytometry.

### Mesothelin sequence analysis

Three different secondary structure prediction servers, nnpredict [35], jpred [36], and 3d-pssm [37], all predicted mesothelin to be made of a series of helices separated by short coil segments, with no beta-sheet content. Three different fold recognition servers, 3d-pssm [37], genthreader [38], and robetta [39], gave hits to proteins which had the alpha-alpha superhelical structure made of the repeating HEAT and ARM motifs [40].

### Homotypic and heterotypic doublet formation assay

The A431-Meso^+^, A431-Meso^-^, #12 and #7 cells were harvested at confluency by trypsin digestion. Cells were washed twice with PBS, then labeled with 1.25 pM CellTracker green (#12 and #7) or 190 pM Cell Tracker Blue (A431-Meso^+ ^and A431-Meso^-^) for 30 min at 37°C. Cells were washed one time with PBS to remove excess dye. The #7 and #12 cells were incubated in the presence or absence of ascites with the A431-Meso^+ ^or the A431-Meso^- ^cells for 1 h on ice. When ascites was not used the incubations were performed in PBS containing 1% BSA. The cell suspensions were gently mixed on a cell shaker. Following incubation, the cells were analyzed by flow cytometry on a LSR-II (BD Biosciences). Vortexing or vigorous shaking of the tubes was avoided prior to flow cytometry measurements. During the flow cytometry measurement, the live cells (cells that excluded propidium iodide) were gated. Data from all of the events, singlet and doublet, was collected for analysis. For heterotypic adhesions, the percentage of events staining for both the blue and the green dyes were analyzed. Live homotypic adhesions were measured using the width parameter on forward scatter.

## Competing interests

The author(s) declare they have no competing interests.

## Authors' contributions

JG designed and conducted a majority of the experiments and helped in the production of this manuscript. JB performed some of the flow cytometry experiments. MO developed the anti-mesothelin antibodies. CR and MM developed the MUC16 knock-down cells. MH and TB developed the mesothelin transfected A431 cells. JC was involved in the design of the experiments and also obtained the ascites samples from patients. BKS and BL performed the protein modeling studies. IP was involved in designing the experiments. MSP was also involved in designing the studies and writing this manuscript.

## Supplementary Material

Additional File 1 Mesothelin does not bind to other WGA and E-PHA binding cell surface associated glycoproteins. Erythrocytes from two donors, D2 and D3, were incubated with meso-Fc for one hour and detected with a FITC conjugated GAR secondary antibody (A). Control erythrocytes from D2 incubated with FITC-conjugated GAR are in grey. The OVCAR-3, #12, #9, and #7 were labeled with biotinylated WGA and E-PHA, (B and C, respectively). FITC-conjugated avidin was used to detect binding of the lectins to the cells by flow cytometry. Click here for file

Additional File 2 Doublet formation between MUC16 and mesothelin expressing cells. Average percentage of doublet frequency was calculated as a mean of two independent experiments between A431 + and -, and #7 and #12 cell lines. Click here for file
