# Supplementary figures and images for: Mesothelin-MUC16 binding is a high affinity, N-glycan dependent interaction that facilitates peritoneal metastasis of ovarian tumors
Source: Mol Cancer. 2006 Oct 26;5:50. doi: 10.1186/1476-4598-5-50 (PMC1635730; doi:10.1186/1476-4598-5-50)

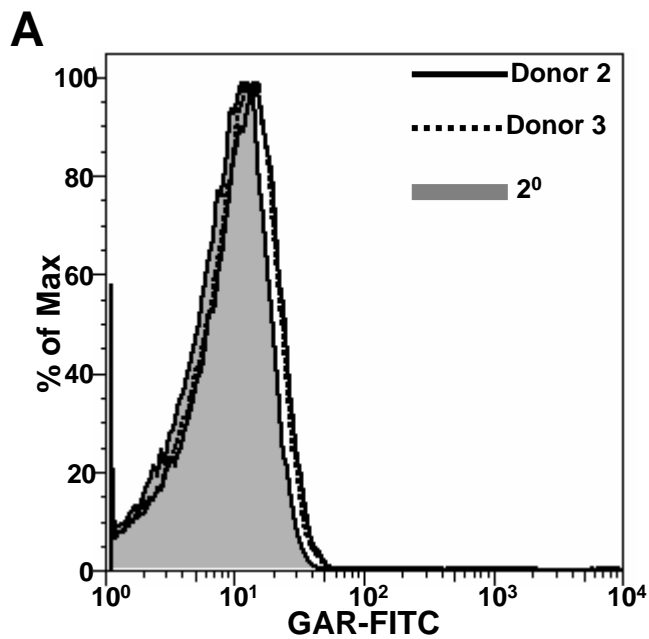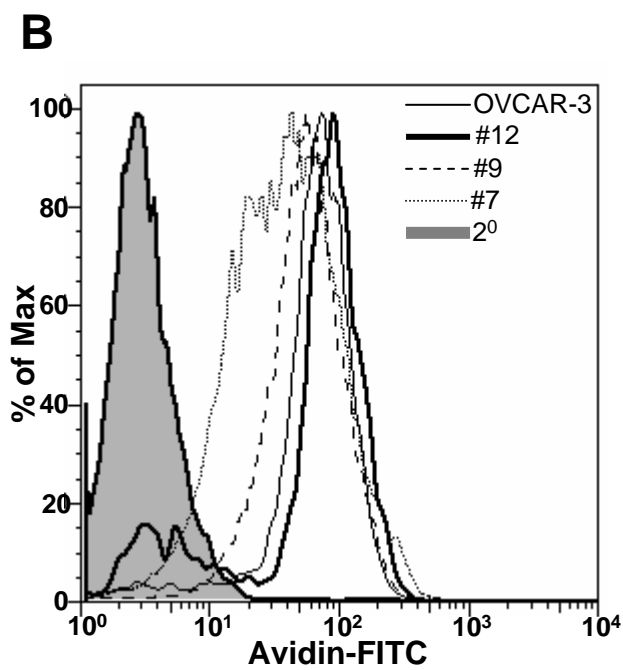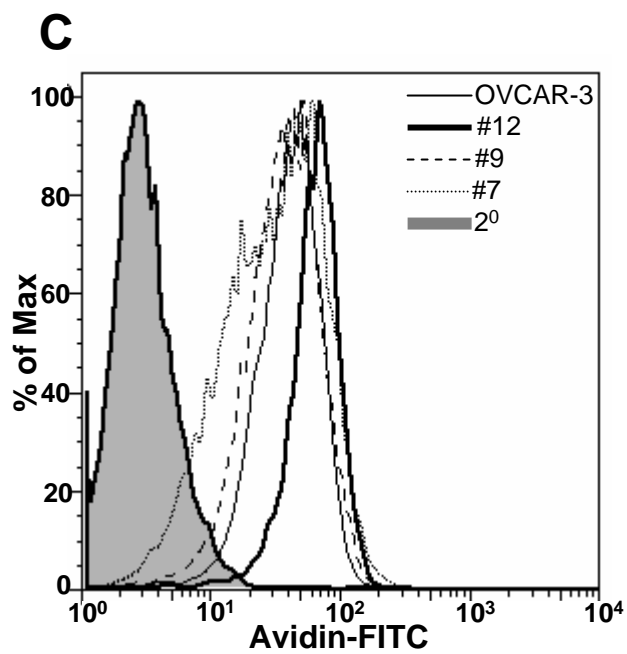

Supplement: Additional File 1 — Mesothelin does not bind to other WGA and E-PHA binding cell surface associated glycoproteins. Erythrocytes from two donors, D2 and D3, were incubated with meso-Fc for one hour and detected with a FITC conjugated GAR secondary antibody (A). Control erythrocytes from D2 incubated with FITC-conjugated GAR are in grey. The OVCAR-3, #12, #9, and #7 were labeled with biotinylated WGA and E-PHA, (B and C, respectively). FITC-conjugated avidin was used to detect binding of the lectins to the cells by flow cytometry. [file 1476-4598-5-50-S1.pdf]
