# Supplementary material for: Mesothelin-MUC16 binding is a high affinity, N-glycan dependent interaction that facilitates peritoneal metastasis of ovarian tumors
Source: Mol Cancer. 2006 Oct 26;5:50. doi: 10.1186/1476-4598-5-50 (PMC1635730; doi:10.1186/1476-4598-5-50)
Supplement: Additional File 2 — Doublet formation between MUC16 and mesothelin expressing cells. Average percentage of doublet frequency was calculated as a mean of two independent experiments between A431 + and -, and #7 and #12 cell lines. [file 1476-4598-5-50-S2.pdf]

Cell Combinations	Suspension Conditions	Average Percentage of Doublet Frequency*
#7 + A431-Meso-	1% PBS/BSA	0.8
#7 + A431-Meso+	1% PBS/BSA	1.5
#12 + A431-Meso-	1% PBS/BSA	8.5
#12 + A431-Meso+	1% PBS/BSA	**25.2**
#7 + A431-Meso-	Pt. 15 ascites	1.4
#7 + A431-Meso+	Pt. 15 ascites	1.2
#12 + A431-Meso-	Pt. 15 ascites	4.0
#12 + A431-Meso+	Pt. 15 ascites	**16.7**
#7 + A431-Meso-	Pt. 24 ascites	1.1
#7 + A431-Meso+	Pt. 24 ascites	0.9
#12 + A431-Meso-	Pt. 24 ascites	4.4
#12 + A431-Meso+	Pt. 24 ascites	**17.1**

***Data in each row is an average of two independent experiments**
